# Cytoplasmic p21 Mediates 5-Fluorouracil Resistance by Inhibiting Pro-Apoptotic Chk2

**DOI:** 10.3390/cancers10100373

**Published:** 2018-10-09

**Authors:** Arnatchai Maiuthed, Chuanpit Ninsontia, Katharina Erlenbach-Wuensch, Benardina Ndreshkjana, Julienne K. Muenzner, Aylin Caliskan, Ahmed P. Husayn, Chatchai Chaotham, Arndt Hartmann, Adriana Vial Roehe, Vijayalakshmi Mahadevan, Pithi Chanvorachote, Regine Schneider-Stock

**Affiliations:** 1 Department of Pharmacology and Physiology, Faculty of Pharmaceutical Sciences, Chulalongkorn University, Bangkok 10330, Thailand; m.arnatchai@gmail.com (A.M.); chuanpit.nin@gmail.com (C.N.); 2 Experimental Tumor Pathology, University Hospital of Friedrich-Alexander University Erlangen-Nürnberg (FAU), 91054 Erlangen, Germany; Benardina.Ndreshkjana@uk-erlangen.de (B.N.); Julienne.Muenzner@uk-erlangen.de (J.K.M.); aylin.caliskan@uk-erlangen.de (A.C.); 3 Institute of Pathology, University Hospital of Friedrich-Alexander University Erlangen-Nürnberg (FAU), 91054 Erlangen, Germany; Katharina.Erlenbach-Wuensch@uk-erlangen.de (K.E.-W.); Arndt.Hartmann@uk-erlangen.de (A.H.); 4 Institute of Bioinformatics & Applied Biotechnology (IBAB), Bangalore 560100, India; husaynahmed@gmail.com (H.A.P.); viji.mv@gmail.com (V.M.); 5 Department of Biochemistry and Microbiology, Faculty of Pharmaceutical Sciences, Chulalongkorn University, Bangkok 10330, Thailand; cchoatham@gmail.com; 6 Department of Pathology, Federal University of Health Sciences of Porto Alegre (UFCSPA), Porto Alegre 90050-170, Brazil; aroehe@gmail.com

**Keywords:** 5-fluorouracil resistance, p21, cytoplasmic p21, Chk2, colorectal cancer, protein interaction

## Abstract

The oncogenic cytoplasmic p21 contributes to cancer aggressiveness and chemotherapeutic failure. However, the molecular mechanisms remain obscure. Here, we show for the first time that cytoplasmic p21 mediates 5-Fluorouracil (5FU) resistance by shuttling p-Chk2 out of the nucleus to protect the tumor cells from its pro-apoptotic functions. We observed that cytoplasmic p21 levels were up-regulated in 5FU-resistant colorectal cancer cells in vitro and the in vivo Chorioallantoic membrane (CAM) model. Kinase array analysis revealed that p-Chk2 is a key target of cytoplasmic p21. Importantly, cytoplasmic form of p21 mediated by p21^T145D^ transfection diminished p-Chk2-mediated activation of E2F1 and apoptosis induction. Co-immunoprecipitation, immunofluorescence, and proximity ligation assay showed that p21 forms a complex with p-Chk2 under 5FU exposure. Using in silico computer modeling, we suggest that the p21/p-Chk2 interaction hindered the nuclear localization signal of p-Chk2, and therefore, the complex is exported out of the nucleus. These findings unravel a novel mechanism regarding an oncogenic role of p21 in regulation of resistance to 5FU-based chemotherapy. We suggest a possible value of cytoplasmic p21 as a prognosis marker and a therapeutic target in colorectal cancer patients.

## 1. Introduction

5-Fluorouracil (5FU) is one of the most commonly prescribed drugs for treatment of various solid tumors, especially colorectal cancer (CRC) [1,2]. However, 5FU resistance is known to be a major obstacle for efficient 5FU-based chemotherapy in cancer patients [1,2,3]. The response rates of metastatic CRC for 5FU single treatment and combinations with other agents are about 15% and 50%, respectively [1,3]. Therefore, new strategies of cancer treatment are urgently needed.

The p21 protein (also known as WAF1 or CIP1) was initially documented to be a tumor suppressor protein due to its ability to inhibit cyclin-dependent kinase (CDK) and proliferating cell nuclear antigen (PCNA) functions, finally contributing to cell cycle arrest [4,5,6]. However, accumulating evidence has suggested that p21 may also play an oncogenic role. In this regard, high expression levels of p21 in many cancer types are correlated with tumor progression [7,8,9,10,11]. A phase I clinical study showed that the potentiation of irinotecan by flavopiridol is due to the reduction of p21 [12]. Likewise, the induction of p21 in rectal carcinoma is associated with resistance to many therapeutic regimens [13]. So far, there is no distinct mechanism that sufficiently explains the dual functions of p21. It was suggested that the role of p21 on cell function control may depend on its sub-cellular localization. Tumor-suppressive functions of p21 are rather associated with a nuclear localization, while more oncogenic activities are found when p21 is localized in the cytoplasm [4,14]. Dependent on its subcellular localization, p21 promotes apoptosis in response to different stimuli [4,14,15,16] or rather triggers pro-survival signaling [17,18,19,20,21,22], which might then contribute to cancer progression and chemotherapeutic resistance. As an example, cytoplasmic p21 has been shown to mediate cisplatin and paclitaxel resistance in various cancers [23,24,25].

Only a few reports describe potential mechanisms of cytoplasmic retention of p21. In general, the nuclear localization signal (NLS) and the nuclear export signal (NES) motifs are responsible for the active shuttling of proteins between the nucleus and the cytoplasm. Thus, the accessibility of the NLS and NES regions under different stress conditions could determine the final localization of p21. Moreover, since the NLS of p21 is localized in close proximity of phosphorylation sites, it is suggested that kinases are responsible for the compartment switch of p21. For example, activated AKT is able to phosphorylate p21 at threonine 145 (T^145^), which results in a cytoplasmic localization of p21 [26]. Indeed, an overactivation of AKT kinase signaling has been shown in 5FU-resistant colon cancer cells [27]. Cytoplasmic p21 also interacts with Rho kinase 2, an inhibitor of stress fiber formation and ASK1 to differentiate myofibroblasts into fibroblasts and to protect them from apoptosis [28]. Thus, altered nuclear export mechanisms or inhibition of nuclear translocation of p21 could be responsible for cytoplasmic localization of p21 under drug exposure. Meanwhile, the general importance of the cytoplasmic–nuclear transport machinery for cancer has been accepted and it was recently proposed as a promising target for anticancer therapy [29]. Nevertheless, the exact mechanism, by which cytoplasmic p21 mediates chemoresistance and which interaction partners are involved, remains majorly unknown. Especially the role of cytoplasmic p21 in controlling 5FU resistance of CRC has never been addressed. Our present study has identified pChk2^T68^ as a novel interaction partner of cytoplasmic p21 that seems to confer to 5FU resistance in CRC. We could verify interplay between p21 and pChk2^T68^ not only in vitro, but also in vivo and in silico.

## 2. Results

### 2.1. Cytotoxic Effect of 5FU on Colorectal Cancer Cell Lines

**Figure 1:**
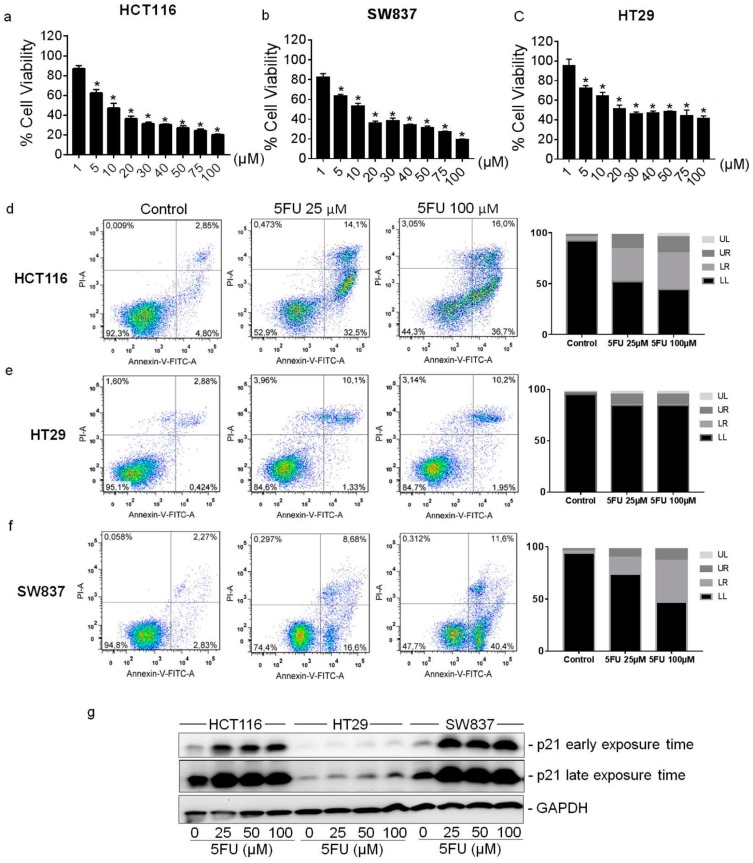
Susceptibility of colorectal cancer cell lines towards 5FU treatment and p21 expression in 5FU-resistant cells. Effects of 5FU on cell viability of (**a**) HCT116 (**b**) SW837 and (**c**) HT29 cells. Cells were treated with various concentrations of 5FU (0–100 μM) for 48 h. The percentage of cell viability was determined by the MTT assay. Values represent means ± SD of three independent experiments. * *p* < 0.05 versus non-treated control. Effects of 5FU on cell apoptosis of (**d**) HCT116 (**e**) HT29 and (**f**) SW837 cells. Cells were treated with 25 μM or 100 μM of 5FU for 48 h. Apoptotic cell death was determined by Annexin-PI co-staining and fluorescent signals were analyzed by flow cytometry. UL: upper left (necrosis), LL: lower left (vital), LR: lower right (apoptosis), UR: upper right (late apoptosis). (**g**) The expression level of p21 in 5FU-resistant cells was determined by Western Blot analysis in three colorectal cancer cell lines. Cells were treated with various concentrations of 5FU for 48 h. After incubation, dead cells were discarded by washing the plates 3 times with PBS, and the remaining resistant cells were collected to prepare protein lysates as mentioned in the Material and Methods section. The blots were re-probed with GAPDH to confirm equal loading of the samples.

To define a suitable experimental model for investigation of the role of p21 for 5FU resistance, we evaluated the susceptibility to 5FU treatment in three different colorectal tumor cell lines (HCT116, HT29 and SW837). MTT assay showed the reduction of cell viability in a dose-dependent manner after 48 h of 5FU treatment in all investigated cell lines. IC50 values were 10 μM for HCT116 and SW837 cells (Figure 1a,b) and 20 μM for HT29 cells (Figure 1c). Consistently, Annexin-PI flow cytometric analysis indicated that the number of apoptotic cells was also increased in a dose-dependent manner for HCT116 and SW837 cells, but not for the rather resistant HT29 cells (Figure 1d–f). Although the endogenous p21 level differed between the three cell lines, there was a remarkable increase in p21 in adherent resistant HCT116 and SW837 cells, whereas the resistant HT29 cells increased their p21 expression to a much lower extent after 5FU exposure, possibly due to their mutant p53 status (Figure 1g). Thus, our results suggest a potential involvement of p21 in reduced response to 5FU in CRC cells.

### 2.2. Cytoplasmic Localization of p21 in 5FU-Resistant Cells In Vitro and In Vivo

**Figure 2:**
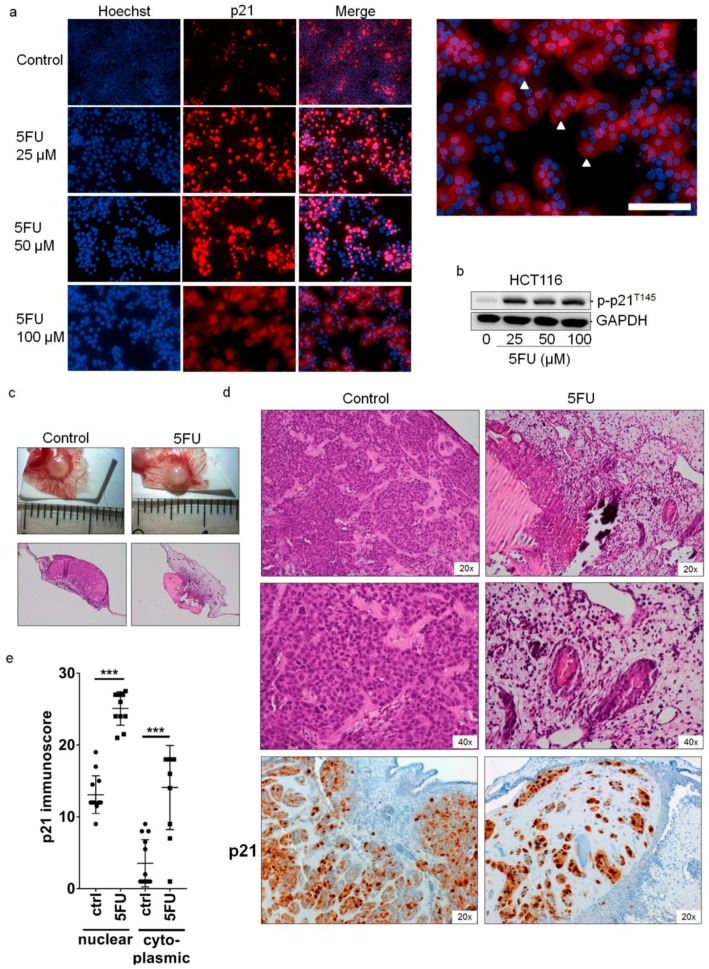
Localization of p21 in 5FU-resistant HCT116 cells. (**a**) HCT116 cells were treated with various concentrations of 5FU for 48 h and the expression of p21 was determined by immunofluorescence staining using mouse anti-p21 monoclonal antibodies followed by an Alexa Fluor 555-labeled secondary antibody to visualize p21 expression (red) and the nuclei (Hoechst 33342, blue). Scale bar: 50 μm. (**b**) The expression level of phosphorylated-p21 (p-p21^T145^) in 5FU-resistant HCT116 cells was determined by Western Blot analysis. Cells were treated with various concentrations of 5FU for 48 h. After incubation, dead cells were discarded by washing the plates 3 times with PBS and the remaining resistant cells were collected to prepare protein lysates as mentioned in the Material and Methods section. The blots were re-probed with GAPDH to confirm equal loading of the samples. (**c**) *Ex ovo* images and overviews of H&E-stained sections of CAM micro-tumors. For this, HCT116 cells were treated with 15 μM of 5FU for 48 h. Then the 5FU-resistant HCT116 cells were subjected to the CAM. After 5 days, tumor tissues were collected, tumor size was measured, and xenografts were subjected to standard histological and immunohistochemical procedures. (**d**) H&E staining and immunohistochemical staining of p21 protein in vital areas of tumor slices. (**e**) Immunoscore of p21 regarding its cytoplasmic and nuclear localization in xenografts of 5FU-treated HCT116 and control cells. *** *p* < 0.001 versus non-treated control.

Next, we investigated the subcellular localization of p21 in CRC cells exposed to 5FU by immunofluorescence. After 48 h of 5FU treatment, dead cells were removed by washing with PBS and the remaining adherent (mainly resistant) cells were stained with a p21 antibody. Figure 2a clearly indicates that p21 was mainly expressed in the cytoplasm of HCT116 cells and this cytoplasmic p21 increased in a dose-dependent manner. Some studies have reported that AKT-driven phosphorylation of p21 (p-p21^T145^) is majorly responsible for p21 accumulating in the cytoplasm [25,26]. However, as the applied p-p21^T145^ antibody produced a high number of unspecific bands in Western Blots (Figure S1, Supplementary Materials), it could not be used for immunofluorescence analysis. Nevertheless, in Western Blot experiments, the correct band of p-p21^T145^ could be identified by loading a sample of HCT116 p21−/− cells (Figure S1, Supplementary Materials). Here, we found a dose-dependent increase in expression of p-p21^T145^ in 5FU-treated cells (Figure 2b). Consistently, similar results were obtained for HT29 and SW837 cells (Figure S2, Supplementary Materials) and the more resistant HT29 cells exhibited the highest endogenous p-p21^T145^ expression level. Next, we performed in vivo xenograft experiments using the CAM assay. HCT116 cells were treated with 5FU for 48 h, the supernatant with apoptotic dead cells was removed, and 1 × 10^6^ cells were then transplanted onto the CAM, and tumor xenografts were harvested after 5 days of inoculation (13× control and 11× 5FU pre-treated specimens, Figure 2c). 5FU treatment led to remarkable reductions in tumor size (22 mm^3^ to 9.4 mm^3^) and vital tumor cell area (86.2% to 11%), and an increase in necrotic areas (4.2% to 23%) (Figure 2d: representative images are shown). In the vital cells of the micro-tumors, which were expected to represent the 5FU-resistant cell population, we could observe an increase in p21 expression in the nucleus and cytoplasm after 5FU treatment (Figure 2d,e).

### 2.3. Cytoplasmic p21 Induces Apoptosis Resistance after 5FU Treatment

**Figure 3:**
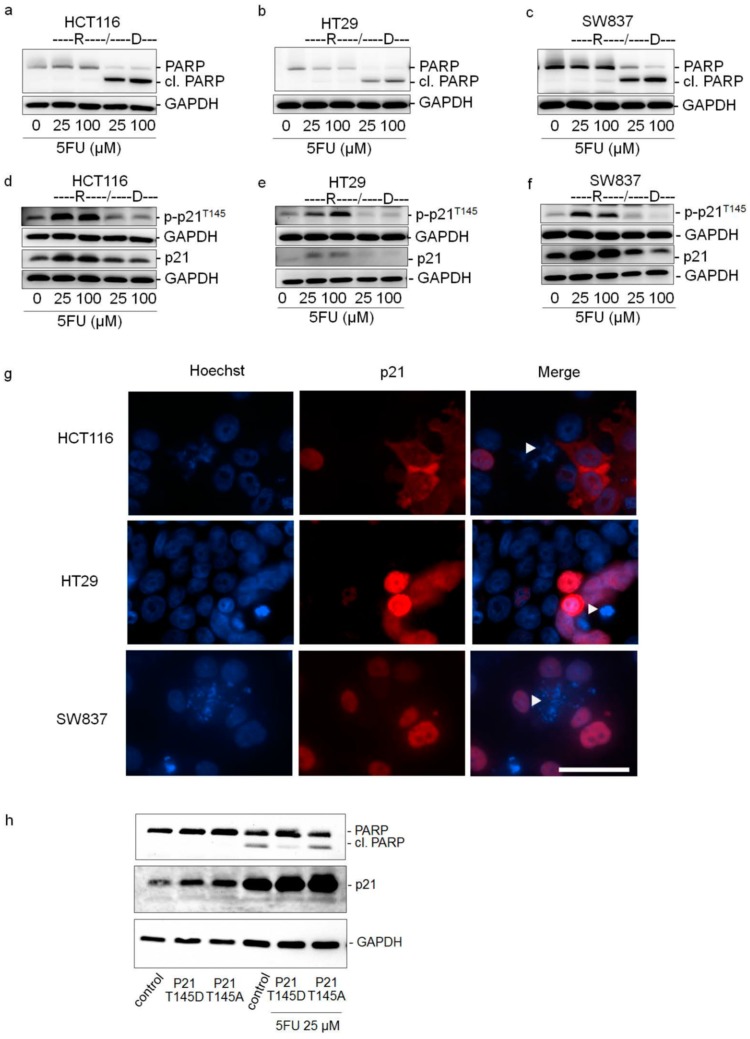
Cytoplasmic p21 mediates 5FU resistance in colorectal cancer cells. (**a**–**f**) The expression levels of PARP, cleaved PARP (cl. PARP), phosphorylated-p21 (p-p21^T145^) and p21 in resistant cells (R) and dead cells (D) were determined by Western Blot analysis after 48 h of treatment with 5FU. For this, HCT116, HT29, and SW837 cells were treated with various concentrations of 5FU. After 48 h, resistant cells (R) and dead cells (D) were collected separately and protein lysates were prepared. The blots were re-probed with GAPDH to confirm equal loading of the samples. (**g**) Expression of p21 in viable and dead cells after 48 h of 5FU treatment. HCT116, HT29, and SW837 cells were treated with various concentrations of 5FU for 48 h and the expression levels of p21 were determined by immunofluorescence staining for p21 (red) and the cell nuclei (Hoechst 33342, blue). White arrow indicates apoptotic cells having condensed chromatin and/or fragmented nuclei. Scale bar: 50 μm. (**h**) 5FU susceptibility of HCT116 cells transfected with hyperphosphorylated p21^T145D^ and unphosphorylatable p21^T145A^. After 24 h of transfection, cells were treated with 5FU (25 μM) for further 48 h. Untreated cells were used as controls. The expression levels of PARP, cleaved PARP (cl. PARP), and p21 were determined by Western Blot analysis. The blots were re-probed with GAPDH to confirm equal loading of the samples.

To further evaluate the role of cytoplasmic p21 in 5FU resistance, control as well as separately collected resistant (R, adherent) and dead (D, detached) cells after 5FU exposure for 48 h were analyzed for PARP cleavage, p21, and p-p21^T145^ protein expression by Western Blot analysis. PARP is known to be one of the major targets of caspase-3 and PARP cleavage is a commonly applied marker of apoptotic cell death [30,31]. The nearly complete absence of cleaved PARP in resistant adherent cells (R) indicated that low or no apoptotic signaling was activated in these cells. In contrast, high PARP cleavage could be observed in apoptotic floating cells (D) (Figure 3a–c). As anticipated from the CAM experiments, there was a remarkable increase in both total p21 and p-p21^T145^ levels for vital cells (R) of all three cell lines. While the total p21 protein expression decreased to the corresponding control levels in apoptotic cells (D), the p-p21^T145^ protein expression was even reduced below the control levels (Figure 3d–f). Immunofluorescence images of 5FU-treated HCT116, HT29 and SW837 cells confirmed these findings, showing a cytoplasmic enrichment of p21 in viable adherent cells. Interestingly, the few apoptotic cells with signs of chromatin condensation did not show p21 at all (Figure 3g).

Since there was a remarkable cytoplasmic p21 increase under 5FU treatment in the resistant cells in vitro and in vivo, we transfected HCT116 cells with the hyperphosphorylated p21^T145D^ or the non-phosphorylatable p21^T145A^ form of p21. In general, transfection without 5FU treatment did not induce PARP cleavage. However, 5FU treatment led to a strong increase in p21 without PARP cleavage in p21^T145D^-transfected cells, while in control cells and cells transfected with the p21^T145A^ form, PARP cleavage was induced (Figure 3h). Moreover, nuclear and cytoplasmic localizations of p21 after p21^T145D^ transfection were verified by immunofluorescence, whereas p21 after p21^T145A^ transfection was found mostly in the nucleus (Figure S3a, Supplementary Materials). Successful transfection after 24 h is shown in Figure S3b (Supplementary Materials). It should be noted that transient transfection led to a normalization of p21 level after 72 h (Figure 3h, left panel), but cells were treated with 5FU at the time point of maximal p21 expression (Figure S3b, Supplementary Materials).

**Figure 4:**
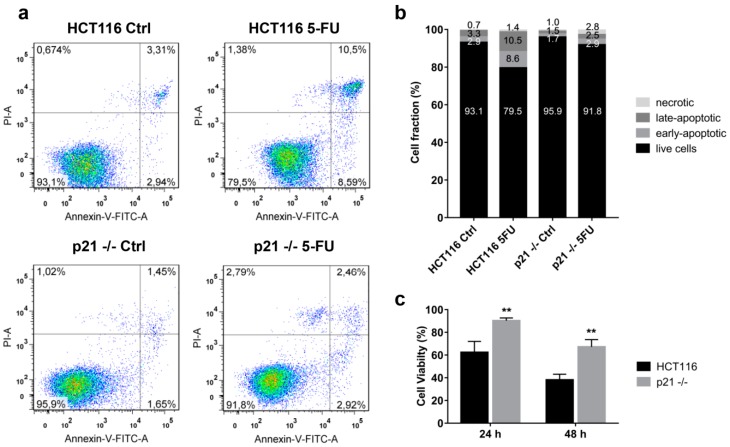
Susceptibility of HCT116 wild-type (HCT116) and HCT116 p21 knockout (p21−/−) colorectal cancer cell lines towards 5FU treatment. (**a**) Effects of 5FU (10 μM) on the induction of apoptosis and necrosis in HCT116 and p21−/− cells after 48 h of incubation as assessed by the Annexin-PI assay. The experiment was carried out in technical and biological duplicate. One representative experiment is shown. (**b**) Fractions of live, early-apoptotic, late-apoptotic and necrotic cells in 5FU-treated and control HCT116 and p21−/− cell populations as determined from the Annexin-PI assay after 48 h of incubation. Values of one representative experiment are shown. (**c**) Cell viability of HCT116 and p21−/− cells after treatment with 5FU (10 μM) for 24 h and 48 h with respect to DMSO controls. Values represent means ± SD of three independent experiments. ** *p* < 0.01.

At this point, from our experimental data obtained from the three CRC cell lines (HCT116, HT29 and SW837) described above, we would expect that HCT116 cells without p21 should be more sensitive to 5FU. To test this, we treated HCT116 p21−/− cells with 10 μM 5FU and performed an Annexin-PI-FACS analysis (48 h, Figure 4a,b) and crystal violet assay (24 h and 48 h, Figure 4c) to determine the viability of cells. Interestingly, p21−/− cells were even more resistant to the 5FU treatment, suggesting that cells without p21 paradoxically take another pathway to develop a resistance to 5FU treatment.

### 2.4. Cytoplasmic p21 Activates Cell Survival Signals and Attenuates the Pro-Apoptotic Effect of Chk2 In Vitro and In Vivo

**Figure 5:**
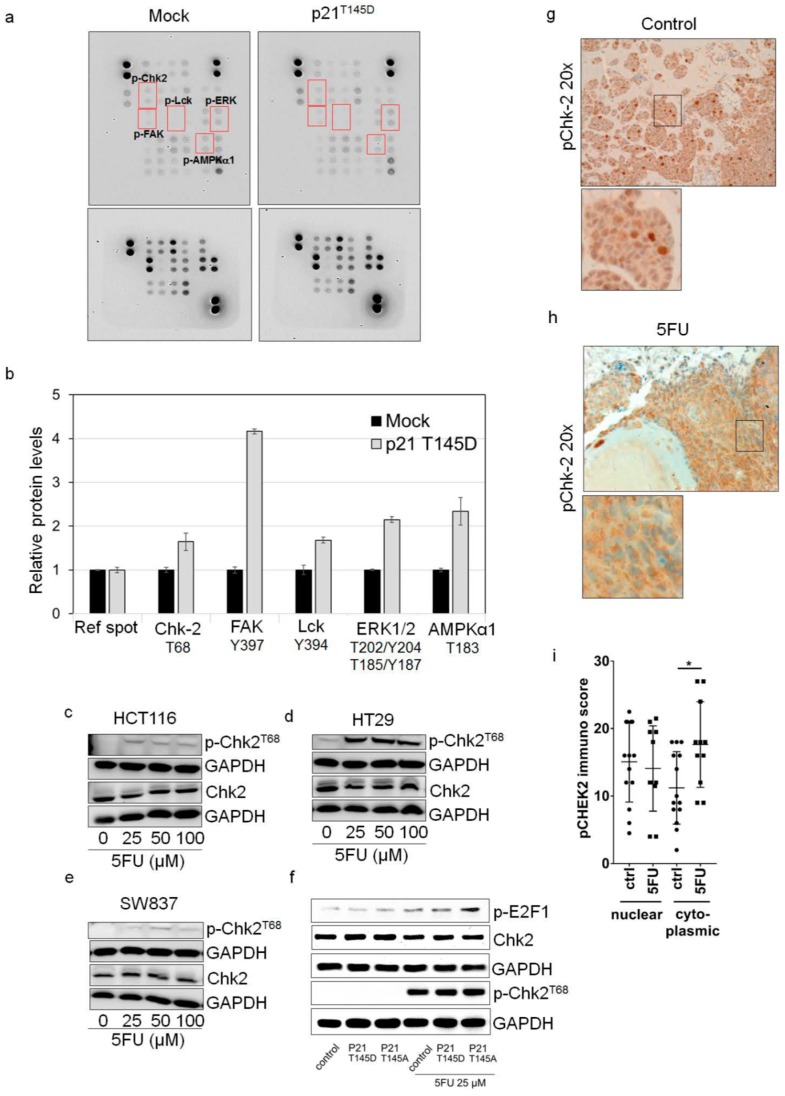
p21 inhibits pro-apoptotic effect of Chk2. (**a**,**b**) Expression levels of phospho-proteins in transfected cells. HCT116 cells were transfected with hyperphosphorylated p21^T145D^. After 48 h of transfection, cells lysates were prepared and subjected to the Human Phospho-Kinase Array Kit (R&D systems). The expression levels of phosphorylated Chk2 (p-Chk2^T68^) and Chk2 in 5FU-resistant HCT116 (**c**), HT29 (**d**), and SW837 (**e**) cells were determined by Western Blot. (The same lysates were loaded on two different membranes for Chk2 and pChk2 detection). For this, cells were treated with various concentrations of 5FU for 48 h. Dead cells were removed by washing with PBS and the remaining viable cells were collected to prepare protein lysates as mentioned in the Material and Methods sections. The blots were re-probed with GAPDH to confirm equal loading of the samples. (**f**) The expression levels of phosphorylated Chk2 (p-Chk2^T68^), Chk2, and p-E2F1 in HCT116 cells transfected with hyperphosphorylated p21^T145D^ and unphosphorylatable p21^T145A^ in response to 5FU treatment were determined by Western Blot analysis. After 24 h transfection, cells were left untreated or were treated with 5FU (25 μM) for further 48 h. Dead cells were removed by washing with PBS and the remaining viable cells were collected to prepare protein lysates. The expression of p-E2F1, p-Chk2^T68^ and Chk2 were determined and the blots were re-probed with GAPDH to confirm equal loading of the samples. Immunohistochemical staining of p-chk2^T68^ in CAM xenografts formed by HCT116 control (**g**) and HCT116 5FU-treated (**h**) cells. (**i**) p-chk2^T68^ immunoscore in cytoplasmic and nuclear localizations in tumors of the HCT116 5FU-treated and untreated groups. * *p* < 0.05 versus non-treated control.

To identify which kinase could be a crucial interaction partner of p21 for 5FU resistance, we quantified the expression of a phospho-kinase panel in control and the hyperphosphorylated p21^T145D^-transfected cells using an antibody-based array. Figure 5a,b shows that there was an increase in phosphorylation of Chk2 (T68), FAK (Y397), Lck (Y394), ERK 1/2 (T202/Y204, T185/Y187), and AMPKα1 (T183) in the hyperphosphorylated p21^T145D^-transfected cells when compared to the control cells. In addition, as it is known that phosphorylation of p21 at threonine 145 is mediated by the activity of AKT, hyperphosphorylated AKT^T308D,S473D^ was used to investigate the expression of human kinase profile after transfection of HCT116 cells with hyperphosphorylated AKT^T308D,S473D^ plasmid. As expected, transfection with hyperphosphorylated AKT^T308D,S473D^ provided the same pattern of up-regulated kinases as transfection with hyperphosphorylated p21^T145D^ (Figure S4, Supplementary Materials).

Thus, for the first time, we identified possible targets of p-p21 that could be involved in mediating 5FU resistance. We further focused on the interplay of p-p21 with the stress-responsive protein Chk2. This protein seemed to be promising since a few studies already indicated a role of p-Chk2^T68^ for the regulation of apoptosis in response to stress signals [32,33]. Moreover, its forkhead-associated (FHA) domain, which typically binds to phospho-threonine residues [34], might interact with threonine 145 of p21. Therefore, we suggest that p-p21^T145^ might interfere with the pro-apoptotic function of p-Chk2^T68^. To study the interaction between p21 and Chk2 in more detail, we first investigated the expression level of p-Chk2^T68^ and its total form in 5FU-resistant cells (vital adherent cells after 5FU treatment). The results indicated that in all three investigated cell lines, an increased protein expression of p-Chk2^T68^ was found in viable 5FU-resistant cells, while the total Chk2 protein level remained mainly unaffected by 5FU treatment (Figure 5c–e).

To clarify if the increase in p-Chk2^T68^ levels is correlated with an increase in phosphorylation of its major target, p-E2F1 [33], we transfected HCT116 cells with hyperphosphorylated p21^T145D^ or the non-phosphorylatable p21^T145A^ and then studied the p-E2F1 levels by Western Blot after 5FU exposure. While the damage sensor p-Chk2^T68^ was commonly induced after 5FU treatment, transfection with the non-phosphorylatable p21^T145A^ form interestingly resulted in a higher p-E2F1 level than transfection with the hyperphosphorylated p-p21^T145D^ (Figure 5f), suggesting that non-phosphorylatable p21^T145A^ form cannot interact with pChk2 to inhibit its signaling.

In a next step, we stained sections of CAM xenografts with a p-Chk2^T68^ antibody. As expected, a significant increase of p-Chk2^T68^ expression in the cytoplasm was observed in vital tumor cells of 5FU-treated xenografts compared to the untreated control group (*p*-value = 0.0173) with only a slight decrease of p-Chk2^T68^ expression in the nucleus of 5FU-treated cells when compared to the untreated controls (Figure 5g–i). Taken together, we suggest that after 5FU treatment, p-p21^T145^ in resistant vital tumor cells could relocate p-Chk2 from the nucleus to the cytoplasm, thus suppressing the recruitment of p-Chk2 to the pro-apoptotic program.

### 2.5. p21 Interacts with p-Chk2 in 5FU-Resistant Cells

**Figure 6:**
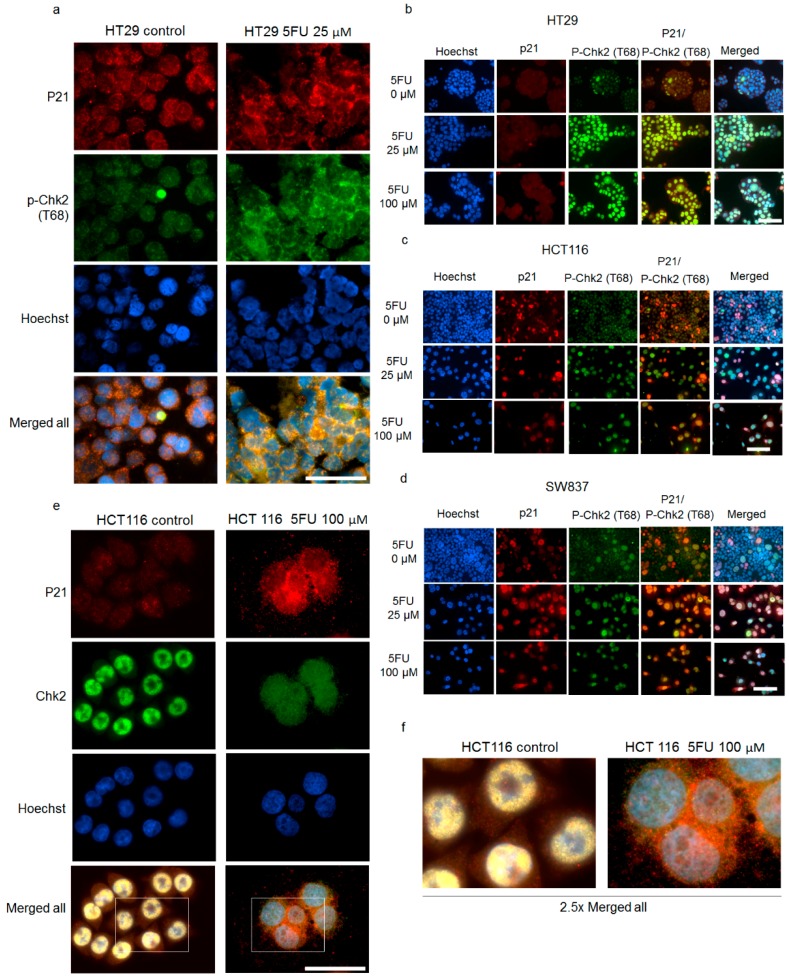
Co-localization of p21 and p-Chk2^T68^/Chk2 in 5FU-resistant cells. (**a**,**b**) HT29, (**c**) HCT116 and (**d**) SW837 cells were treated with various concentrations of 5FU for 48 h and the expression of p21 and p-Chk2^T68^ was examined by immunofluorescence staining using mouse anti-p21 monoclonal antibodies followed by an Alexa Fluor 555-labeled secondary antibody to visualize p21 expression (red), rabbit anti-p-Chk2^T68^ monoclonal antibodies followed by an Alexa Fluor 488-labeled secondary antibody to visualize p-Chk2^T68^ expression (green) and cell nuclei (Hoechst 33342, blue). (**a**) Enlarged images of p21 and p-Chk2T68 co-localization in 5FU-resistant HT29 cells. Scale bar: 50 μm; (**e**) HCT116 cells were treated with 100 μM 5FU for 48 h and the expression of p21 and Chk2 was examined by immunofluorescence staining using mouse anti-p21 monoclonal antibodies followed by an Alexa Fluor 555-labeled secondary antibody to visualize p21 expression (red), rabbit anti-Chk2 monoclonal antibodies followed by an Alexa Fluor 488-labeled secondary antibody to visualize Chk2 expression (green) and cell nuclei (Hoechst 33342, blue). Scale bar: 50 μm; and (**f**) 2.5-fold computer-enlarged images from merge images in (**e**).

**Figure 7:**
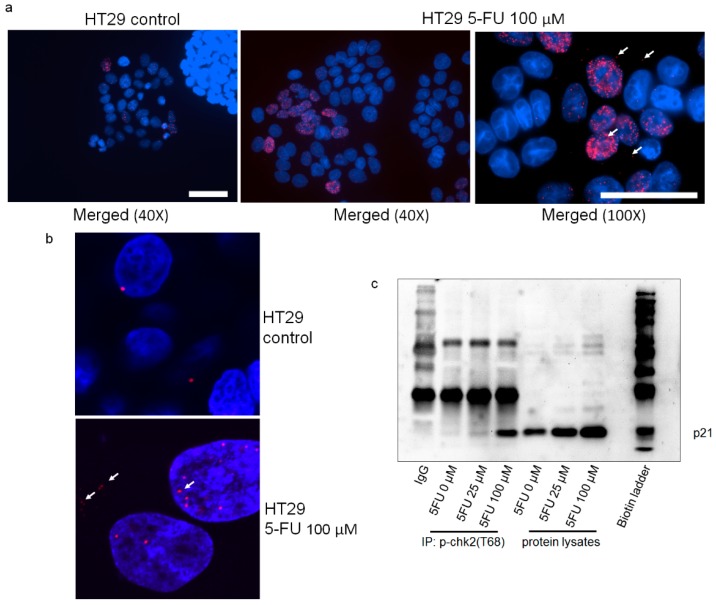

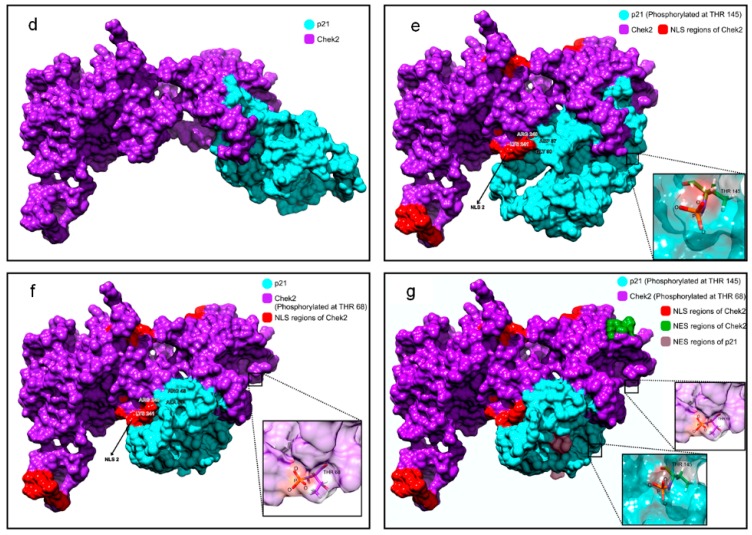
Interaction between p21 and Chk2 proteins. Cells were treated with 100 μM 5FU for 48 h and the protein–protein interaction of p21-p-Chk2^T68^ was analyzed by proximity ligation assay (HT29) (red signals indicate the protein–protein interaction between p21 and p-Chk2^T68^. (**a**) Fluorescence images of untreated control HT29 cells (40× magnification) and 5FU-treated HT29 cells (40× and 100× magnification) (scale bar: 50 μm); (**b**) confocal images. (**c**) 5FU-treated HCT116 cell lysates were prepared and immunoprecipitated with an anti-p-chk2^T68^ antibody. The resulting immune complexes were then analyzed for p21 by Western Blot using an anti-p21 antibody. (**d**) Complex of p21 and Chk2 obtained by structural modelling; p21 (cyan) was modelled using two PDB structures 4I58 and 5EOU as templates using MODELLER [39]. Chk2 (magenta) was obtained from its crystal structure 2WTC after removing the coordinates of the inhibitor. The structures were energy-minimized and docked using ClusPro [40] and rendered using Chimera [40]. (**e**) Complex of phosphorylated p21 and Chek2: the energy-minimized stable structure of p21 (cyan) was phosphorylated at Threonine residue at position 145 using Chimera [41] and was docked with the structural model of Chek2 (magenta) using ClusPro [40]. The nuclear localization signal (NLS) region of Chk2 is shown in red. The insert shows the phosphorylation of T145 of p21 in the model. The interactions between the proteins were identified using the Protein Interaction Calculator (PIC) [42]. (**f**) Complex of p21 and phosphorylated Chk2: the energy-minimized stable structure of Chk2 (magenta) was phosphorylated at Threonine residue at position 68 using Chimera [41] and was docked with the structural model of p21 (cyan) using ClusPro [40]. The NLS region of Chk2 is shown in red and the residues around and in the NLS are labelled. The insert shows the phosphorylation of T68 of Chk2 in the model. The interactions between the proteins were identified using the PIC [42]. (**g**) Complex of phosphorylated p21 (T145) and phosphorylated Chek2 (T68): individual structural models of p21 phosphorylated at T145 and Chek2 phosphorylated at T68 were docked using ClusPro [40] and rendered using Chimera [41]. The NLS of Chek2 is shown in red and the nuclear export signal (NES) is shown in green. The interaction profile of the p-p21/p-Chk2 complex identified by the PIC [42] shows that no amino acid in the NES region of p21 interacts with p-Chk2, hence showing that the NES is free from any interactions facilitating export of the complex to the cytoplasm.

It is well known that p21 directly interacts with several proteins and retains them in the cytoplasm [35,36,37,38]. Thus, we raised the question if p-p21^T145^ and p-Chk2 might be interacting proteins. First, we confirmed a co-localization of p21 and p-Chk2 and p21 and total Chk2 by co-immunofluorescence staining resistant cells after 5FU exposure. Figure 6a–f show that this co-localization was found in both compartments of 5FU-resistant cells in all three cell lines. In a Duo-link assay, we further confirmed a direct physical interaction between p21 and p-Chk2^T68^ mainly in the nuclei and a few signals also in the cytoplasm of 5FU-resistant HT29 cells (Figure 7a). Images of single antibody staining for untreated and 5FU-treated HT29 cells are given in Figure S5a,b in Supplementary Materials. Confocal microscopy images of Duo-link assay are given in Figure 7b. Additionally, we were able to detect an interaction of p21 and p-Chk2^T68^ in 5FU-resistant cells in a dose-dependent manner, performing a co-immunoprecipitation in HCT116 cells (Figure 7c).

**Figure 8:**
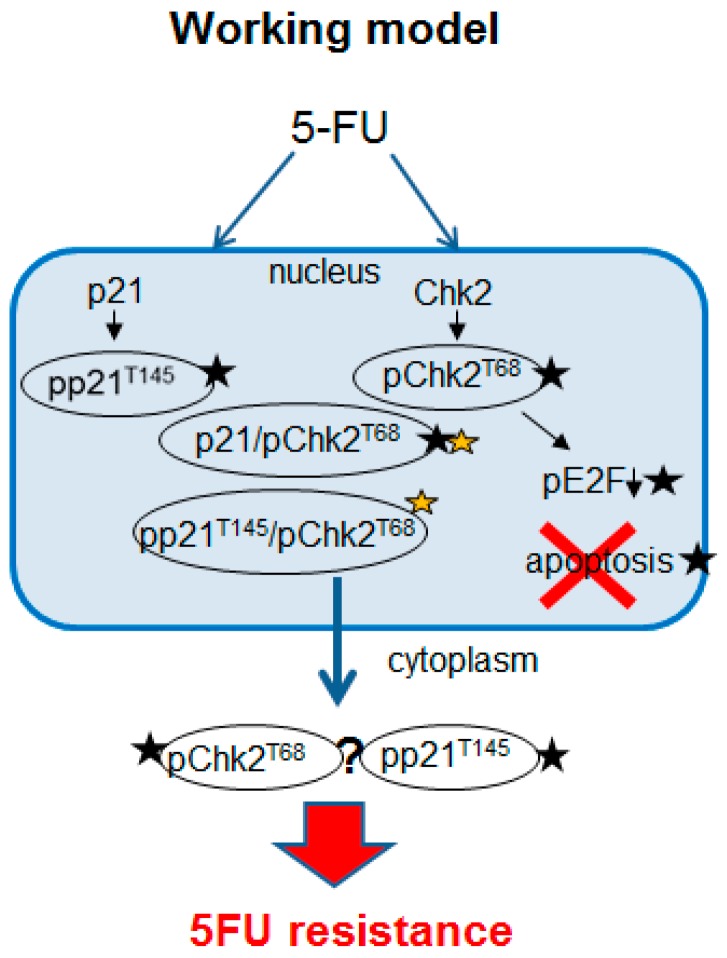
Schematic model of p21/Chk2-mediated 5FU resistance. Black stars mark where experimental evidence is given, and orange star marks in silico analysis. The fate of p-Chk2 in the cytoplasm after detachment of the complex is unclear.

Additionally, the structural modeling presented in Figure 7d–g provides further evidence that p21 has the capability to interact with Chk2. The interaction site of p21 was found in the second NLS of Chk2 at the arginines of positions 240 and 241 (Figure 7e). When the threonine 145 of p21 and the threonine 68 of Chk2 were phosphorylated, the energy of the complex of both phosphorylated molecules was found to decrease from −907.3 kcal/mol to −1083.5 kcal/mol pointing to a stabilized and stronger binding. The schematic model in Figure 8 describes our hypothesis regarding Chk2 localization and function being controlled by p21 in 5FU-treated cells. Phosphorylation at threonine 68 leads to a blockage of the NLS of Chk2 (Figure 7f). We identified two NES sequences of p21 NES1: amino acids 68–78 with the sequence VRGLGLPKLYL and NES2: at amino acids 102–119 with the sequence LQGTAEEDHVDLSLSSCTL. Two NES sequences of Chk2 were found using the prediction tool LocNES: amino acids 92–106 with the sequence PWARLWALQDGFANL and a second one at amino acids 454–468 with the sequence EVSEKALDLVKKLLV with the latter being the relevant NES of Chk2. Examining the interaction profile of the p-p21^T145^/p-Chk2^T68^ complex obtained by structural modeling, it is clearly evident that both NES regions of p-Chk2 are without any interaction with p-p21 facilitating export of the complex to the cytoplasm (Figure 7g). Figure 7g shows the NLS and NES regions in the modeled complex. Since we could not find a prominent cytoplasmic p21/p-Chk2 complex with PLA analysis, we suggest that it might dissociate in the cytoplasm. This in silico model is supported by co-immunoprecipitation data where we can show that Chk2 signals increase with higher 5FU concentrations only when immunoprecipitated with p-p21 but not when immunoprecipitated with the p21 antibody (Figure S6a,b, Supplementary Materials). Thus, the function of p-p21^T145^ might be the permanent export of activated Chk2 from the nucleus, minimizing its pro-apoptotic signaling (Figure 8).

Together, our data demonstrated that interaction of p-p21^T145^ with p-Chk2^T68^ is one of the pro-survival mechanisms contributing to 5FU resistance in CRC cells.

## 3. Discussion

Chemoresistance has become the major limitation for 5FU-based therapy in multiple types of cancer [1,3]. Thus, there is an urgent need to better understand the underlying molecular mechanism and to finally identify novel biomarkers with the potential to predict the patient’s specific drug response. For the first time, our study revealed that cytoplasmic p21 plays a critical role in controlling 5FU resistance in CRC.

As a classical cell cycle inhibitor and tumor suppressor, p21 mediates diverse cellular functions such as DNA repair, cell cycle arrest, differentiation, and apoptosis induction [4,43]. Interestingly, p21 might also act as an oncogene, which also led to the introduction of the name “cancer gene chameleon” [44,45]. The distinct functions of p21 are mainly dependent on its subcellular localization. Whereas nuclear p21 elicits tumor suppressive activities, the cytoplasmic p21 protein has rather oncogenic effects [4,14].

The cytoplasmic localization of p21 can be ascribed to phosphorylation through different kinases at distinct phosphorylation sites, such as Thr-57, Ser-130, Thr-145, Ser-146, and Ser-153 [46]. Activated AKT was suggested to be an important regulator of p21 and phosphorylates p21 at threonine 145 (p-p21^T145^), leading to formation of anti-apoptotic complexes [26]. Since the phosphorylation of p21 at threonine 145 was clearly shown to cause cytoplasmic retention of the protein, our findings led to the notion that cytoplasmic p21 might control the susceptibility to 5FU. Previous studies have already shown that cytoplasmic p21 is mediating drug resistance for cisplatin and paclitaxel in ovarian cancer [23,24,25]. In doxorubicin-chemoresistant breast cancer cells, cytoplasmic re-localization of p21 led to an up-regulation of the anti-apoptotic protein Bcl_xL_ [47].

Indeed, we showed that, in response to 5FU, the protein levels of p-p21^T145^ were up-regulated predominantly in the resistant (adherent) cells when compared to apoptotic (detached) cells. In addition, there was a remarkable cytoplasmic increase/shuttling under 5FU treatment in the resistant cells in vitro and in vital (resistant) areas of in vivo xenografts. When conducting ectopic expression of two different forms of p21, the hyperphosphorylated p21^T145D^ and unphosphorylatable p21^T145A^, we found that the presence of p-p21^T145D^ conferred 5FU resistance, while unphosphorylatable p21 had the opposite effect. In accordance with these findings, apoptosis induction measured by cleaved PARP seemed to be higher in the hypophosphorylated p21^T145A^ group.

Paradoxically, our data provided evidence that the cytoplasmic phosphorylation and sequestration of p21 is not the only pathway by which resistance to 5FU can develop. Thus, our findings support the general knowledge of a dual functionality of p21 action dependent on the genetic context of cells. It has to be mentioned that HCT116 p21−/− cells show another morphologic differentiation and represent a more mesenchymal phenotype [48]. Mesenchymal colorectal tumor cells are well-known for their high chemotherapy resistance [49]. Therefore, we suggest that cells without p21 take another pathway to develop a resistance to 5FU treatment.

It is well accepted that p21 has the capability to interact with several cellular proteins resulting in its diverse functions [3,4,20,21,22,25,43,50]. Cyclin E/A-CDK2 or cyclin D-CDK4/6 complexes were identified as the main interaction partners of p21 being involved in cell cycle regulation [4,43,50]. In addition, binding of p21 to pro-caspase 3 or ASK1 has been shown to play a role in apoptotic signaling inhibition [20,21,22]. In the present study, the expression of a panel of phospho-kinases analyzed by an antibody-based array suggested phosphorylated Chk2 (T68) to be an interaction partner of p21 that is involved in mediating 5FU resistance in CRC. Chk2 is a serine/threonine protein kinase, which can be activated in response to DNA damage [32,33]. It is well established that the Chk2 protein structure contains an FHA domain, which typically binds to phospho-threonine residues of interacting proteins [34]. Using in silico modeling, we provided further evidence that this domain might interact with p21^T145^. The interaction between both proteins becomes even stronger, when both proteins are phosphorylated, which is reflected by the lower energy of the complex. The functions of Chk2 for DNA damage-response are very diverse and include regulation of DNA repair, cell cycle arrest, senescence and apoptosis [32,33,51]. Thus, Chk2 seemed a promising novel candidate that we wanted to study in more detail concerning its interaction with p21 and role in 5FU resistance. In response to stress stimuli, Chk2 is phosphorylated and activated by ATM at Threonine 68 (p-Chk2^T68^) in the nucleus. Activated p-Chk2 then phosphorylates one of its downstream substrates E2F1, which contributes to the induction of apoptosis [33,34]. In the present study, 5FU treatment caused an increase in the p-Chk2^T68^ level, and for the first time, we describe that cytoplasmic p21 or p-p21^T145^ could inhibit the normal function of p-Chk2 in activation of pro-apoptotic p-E2F1.

We provided further evidences for an interaction between p21 and p-Chk2: (1) p-Chk2^T68^ could be co-immunoprecipitated with p21 after 5FU treatment; (2) cytoplasmic p21 is co-localized with p-Chk2^T68^ after 5FU treatment as shown by immunofluorescence and immunohistochemistry both in vitro and in vivo; (3) our proximity ligation assay showed interaction between both proteins in the nucleus, leading to the hypothesis that p21 is responsible for the nuclear export of p-Chk2^T68^; and (4) in silico modeling supported our hypothesis of a p21/Chk2 protein complex formation. According to our working model, p21 and Chk2 exist in a complex in the nucleus. After 5FU treatment, p21 and Chk2 are phosphorylated and the conformation of both proteins triggers the interaction of p-p21^T145^ and p-Chk2^T68^ at the two residues Arg 240 and Arg 241, where the NLS of Chk2 is located leading to a masked NLS. At the same time, the NES sequences of p-p21^T145^ are still accessible, thus facilitating the export of the complex to the cytoplasm. There was a significant cytoplasmic increase of p-Chk2^T68^ in CAM xenografts of cells that had been treated with 5FU. Obviously, p-p21^T145^ seems to decrease the endogenous level of nuclear p-Chk2^T68^ after 5FU stimulus. From our results of the ligation assay, we assume that the complex is falling apart, when p-Chk2^T68^ reaches the cytoplasm. The fate of p-Chk2 in the cytoplasm after detachment of the protein complex is unclear. For B-cell lymphoma, it has been reported that ERK and Chk2 form a cytoplasmic complex and this functional interaction requires Chk2 phosphorylation [52]. We cannot exclude the interaction of p21 with further kinases as the spectrum of deregulated kinases after p21 transfection was remarkable. Moreover, 5FU is also able to induce cancer cell death, independent of p21 signaling [1,3]. Finally, we suggest p-Chk2^T68^´s nuclear export and cytoplasmic accumulation as a novel mechanism for cytoplasmic p21-mediated resistance to 5FU.

## 4. Material and Methods

### 4.1. Cell Lines

Human colorectal HCT116, HT29, SW837 cancer cells were cultured in RPMI 1640 (GIBCO Life Technologies, Loughborough, UK) and HCT p21−/− cancer cells were cultured in DMEM (GIBCO Life Technologies) supplemented with 10% fetal bovine serum (PAN Biotech, Aidenbach, Germany), penicillin (100 U/ML) and streptomycin (100 μg/mL) (PAN Biotech), 1% l-glutamine (PAN Biotech) (for HCT p21−/−) and 1% essential amino acid (GIBCO Life Technologies) (for HCT p21−/−). Cells were mycoplasma free. Cell lines were authenticated using Multiplex Cell Authentication by Multiplexion (Heidelberg, Germany).

### 4.2. Cytotoxicity Assay

Cell viability was determined by MTT or Crystal Violet assay as previously described [53,54]. Briefly, HCT116, HCT116 p21−/−, HT29, and SW837 cells were seeded in 96-well plates and treated with 5FU at various concentrations (0–100 μM) for 24 h or 48 h and then stained with MTT or Crystal Violet. All analyses were performed in at least three independent replicate experiments.

### 4.3. Annexin-Propidium Iodide Apoptosis Assay by Flow Cytometry

Quantification of apoptosis was performed by Annexin-PI staining as previously described [55]. Cells were treated with 5FU (10, 25, and 100 μM) for 48 h. Positive Annexin V staining indicates early apoptotic cells, and propidium iodide-positive cells were used to measure necrotic cells, whereas Annexin V-positive and propidium iodide-positive cells were counted as late apoptotic cells.

### 4.4. Western Blot Analysis

Cells were treated with 5FU (25 and 100 μM) for 48 h. Dead and viable cells were collected separately. Cell lysates were prepared by adding lysis buffer with a protease inhibitor cocktail (Merck KGaA, Darmstadt, Germany) to cell pellets for 90 min on ice. After SDS PAGE, the proteins were transferred onto 0.45 μM nitrocellulose membranes (GE Healthcare Chalfont, St. Giles, UK). After blocking for 1 h in 5% non-fat dry milk in TBST (25 mM Tris-HCl (Carl Roth, Karlsruhe, Germany) pH 7.5, 125 mM NaCl (Carl Roth, Karlsruhe, Germany), and 0.05% Tween 20 (SERVA) membranes were incubated with a primary antibody at 4 °C overnight. Membranes were washed three times with TBST for 5 min and incubated with horseradish peroxidase-labeled isotype-specific secondary antibodies (anti-mouse or anti-rabbit IgG peroxidase conjugated; Pierce, Rockford, IL, USA) for 2 h at room temperature. The immune complexes were detected by enhancement with a chemiluminescent substrate (IMerck Millipore, Molsheim, France). The level of immunoreactivity was measured as peak intensity using an image capture and analysis system (Syngene Europe, Cambridge, UK). Antibodies used in the present study were as follows: mouse monoclonal anti-p-p21^T145^ (1:1000, BSA, Santa Cruz Biotechnology, Dallas, TX, USA), mouse monoclonal anti-p21 (1:1000, BSA, Cell Signaling), Rabbit monoclonal anti-Chk2 (1:1000, BSA, Cell Signaling, Cambridge, UK), Rabbit monoclonal anti-P-Chk2^T68^ (1:1000, BSA, Cell Signaling), mouse monoclonal p-E2F1 (1:250, BSA, Abcam, Cambridge, UK) and Rabbit monoclonal anti-PARP (1:1000, BSA, Cell Signaling). HRP-conjugated anti-GAPDH (1:50.000, BSA, Abnova, Taipei, Taiwan) was used to control equal loading and protein quality. In addition, lysates from HCT116 p21−/− cells were used as a validation control for the p-p21 antibody.

### 4.5. Immunofluorescence

Cells were treated with 5FU (25 and 100 μM) for 48 h. After the treatment, cells were washed several times with PBS to remove the dead cells. The remaining cells on the slides were fixed with 4% paraformaldehyde for 20 min and permeabilized with 0.1% Triton-X for 10 min. Thereafter, the cells were incubated with 3% bovine serum albumin (BSA) (Carl Roth) for 30 min to prevent nonspecific binding. The cells were washed and incubated with mouse anti-p21 monoclonal antibodies or rabbit anti-p-Chk2^T68^ monoclonal antibodies (1:400 and 1:200 in BSA, respectively) for 48 h at 4 °C. The primary antibody was removed and cells were washed with PBS and subsequently incubated with secondary antibodies (Alexa Fluor 555-labeled conjugated goat anti-mouse IgG or Alexa Fluor 488-labeled conjugated goat anti-rabbit IgG, respectively, 1:500, Invitrogen, Carlsbad, CA, USA) for 2 h at room temperature. Nuclei were counterstained with Hoechst 33342 (Sigma-Aldrich, St. Louis, MO, USA). Samples were washed with PBS, and then visualized and imaged by fluorescence microscope.

### 4.6. Plasmids and Transfection

Flag p21 T145D (Addgene plasmid # 16242), Flag p21 T145A (Addgene plasmid # 16241) and HA PKB T308D S473D pcDNA3 (Addgene plasmid # 14751) were provided by Addgene (Cambridge, MA, USA). Transient transfection was performed using Lipofectamine 3000 Transfection Reagent according to the manufacturer’s instruction (Thermo Fisher Scientific, Waltham, MA, USA). Briefly, HCT116 cells were seeded to be 70–90% confluent at transfection. Plasmid DNA-lipid complexes were prepared as recommended from the company’s protocol. DNA-lipid complexes were added to the cells. Cells were incubated for 2 days at 37 °C before treatment with 5FU (25 μM) for 48 h and evaluation of protein expression by Western Blot analysis. The transcription efficiency was validated by immunofluorescence and Western Blot analysis of targeting gene expression in three independent experiments.

### 4.7. Immunoprecipitation

HCT116 were seeded in 60-mm cell culture dishes at a density of 5 × 10^5^ cells/dish for 24 h. Then, the cells were treated with 5FU (25 and 100 μM) for 48 h. After removing the dead cells, cell lysates of remaining cells were collected and prepared as previously described [56]. Briefly, cells were fixed with 2% formaldehyde before lysed with RIPA buffer. Protein content was determined by using a Bio-Rad Dc Protein Assay (BioRad, Hercules, CA, USA). Equal amounts of lysates (600–900 μg) were adjusted to 500 μL of volume and then incubated with specific antibodies to p-Chk2(T68) immobilized on 50 μL of magnetic Protein G beads overnight at 4 °C (IP Dynabeads Kit). The protein–antibody–bead complexes were recovered (IP Dynabeads Kit) and the protein complexes were analyzed by Western Bloting.

### 4.8. Human Phospho-Kinase Antibody Array

To determine levels of phospho-kinases at baseline, and after specific transfection, HCT116 were harvested after 48 h of transfection with Flag p21 T145D pcDNA3 or HA PKB T308D S473D pcDNA3. Cells were lysed using lysis buffer of the Human phospho-kinase array kit (ARY003, Proteome Profiler^TM^, R&D Systems, Minneapolis, MN, USA). The Human phospho-kinase array was performed according the protocol of the manufacturer. In this array, 46 capture antibodies were spotted in duplicate on nitrocellulose membranes. Cell lysates were incubated with the membrane overnight and membranes were incubated with a cocktail of biotinylated detection antibodies and streptavidin-HRP. Finally, proteins were detected using an ECL chemiluminescent system. The level of immunoreactivity was measured as peak intensity using an image capture and analysis system (GeneGnome). The intensity of signal of each spot was quantified using ImageJ software. The intensity values were corrected for background signals and to compare different membranes levels were normalized to those of the positive controls on each membrane.

### 4.9. Chorioallantoic Membrane Assay (CAM Assay)

The CAM assay represents a well-known and accepted alternative xenograft animal model system. Thus, the present study complies with the commonly accepted “3Rs”. Briefly, fertilized chicken eggs were incubated at 37 °C with a 80% humidity for 8 days. Then, a small hole was pricked into the eggshell of the more rounded pole, where the air sac resided. After dropping of the egg content, a small window (Ø 1–1.5 cm) was carefully cut at the more rounded pole for inoculation of the tumor cells. After another day of incubation (day 9 of embryonic development), 1 × 10^6^ of 5FU pretreated (15 μM) or control HCT116 cells in a 1:1 mixture of Matrigel (Corning Life Sciences, NY, USA) and the medium were implanted onto the CAM and the eggs were incubated for another 5 days. Finally, micro-tumors were harvested, fixed in 4% phosphate-buffered formalin and embedded in paraffin for histological and immunohistochemical analysis.

### 4.10. Immunohistochemistry (IHC) Staining Assay

Serial sections (2 μm) were cut from the paraffin blocks and mounted on pre-coated slides for immunohistochemical analysis of the CAM tumors. All FFPE CAM tissue sections were deparaffinized with xylene and rehydrated with graded ethanol. The antigen retrieval was performed by 1 min steam cooking in TRS-Buffer pH 6 (p21 Waf1/Cip1) or pH 9 (pChk2^T68^), respectively. Slides were incubated at 4 °C overnight with a primary monoclonal antibody p21 Waf1/Cip1 (12D1, mouse), 1:1.000 dilution (Cell Signaling), or pChk2^T68^ (2197, rabbit), 1:50 dilution (Cell Signaling). Antibody binding was visualized using the Polymer-Kit (AP, Zytomed, Berlin, Germany) or biotinylated Anti-Rabbit/ABC-Kit (Vector, Olean, NY, USA).

Adjacent slides were stained with hematoxylin and eosin for histomorphological analyses. Immunohistochemical scoring was performed in a semi-quantitative way by two pathologists (KEW, AVR). The intensity was quantified in a range from 1 to 3. The area was quantified by percentage of positive cells in 5% steps. An immunoscore was generated by multiplying intensity (0–3) with the respective percentage of positive cells (0–100%).

Bright field images (Magnification: 200×, 400×) of stained sections were taken with the Olympus XC50 camera (Olympus Corporation, Tokyo, Japan) in combination with the Olympus BX51 microscope (Olympus Corporation).

### 4.11. Proximity Ligation Assay (In Situ PLA)

HT29 cells were grown on 8-well chamber slides until 70–80% confluency and treated with 5FU. Cells were then washed with PBS, fixed in 4% paraformaldehyde in PBS for 30 min, permeabilized in 0.1% Triton X-100 for 20 min, and blocked with a Duolink II blocking solution (Sigma-Aldrich). After blocking, cells were incubated with a combination of primary antibodies anti-p21 and anti-p-Chk2 (T68) in a preheated humidity chamber for 1 h at 37 °C. Cells were then incubated with the PLA probes diluted 1:5 in antibody diluent (Sigma-Aldrich) in a humidified chamber for 1 h at 37 °C. Subsequent hybridization, ligation, amplification, and detection were performed using manufacturer’s instruction (Sigma-Aldrich). Fluorescence images were acquired using a confocal laser microscope (LSM-PMT Observer, Carl Zeiss, Oberkochen, Germany).

### 4.12. Structural Modeling

Modelling of p21: The full length structure of the 164aa long p21 protein was modelled using two crystallographic structures 4I58 and 5EOU as templates, respectively, for homology by MODELLER [39] and a structure prediction approach using Iterative Threading ASSEmbly Refinement (ITASSER), a hierarchical server for protein structure prediction [57].

For modelling of Chk2, its crystal structure with an inhibitor was taken from the PDB (2WTC) and the coordinates of the inhibitor were then removed to get the structure of Chk2. The structure was energy-minimized and the stable structure was considered for further investigations. Phosphorylation of p21 at T145 and of Chk2 at T68 was modelled using CHIMERA [41]. The complexes of p21 and Chk2 were formed by docking the individual proteins using the protein–protein docking tool ClusPro [40]. The complexes with the lowest energy were chosen for the analysis of interactions between the proteins considered. The cluster scores of the complexes from the ClusPro server were used to understand the energy profiles of the p21–Chk2 complex. The ionic, hydrophobic, and hydrogen bond interactions were identified and analyzed, applying the Protein Interaction Calculator (PIC) [42]. All renderings were performed using CHIMERA [41]. The same protocol was followed for docking of the phosphorylated structures of p21 and Chk2.

### 4.13. Statistics

Data were expressed as means ± SD of at least three independent experiments. All treatment data were normalized to non-treated controls or transfection controls. Statistical analysis was performed using the non-parametric Mann–Whitney test. Statistical significance was set to *p* < 0.05.

## 5. Conclusions

In summary, our mechanistic study provides novel information on how cytoplasmic p21 controls 5FU resistance in CRC. We suggest that the p21-dependent inhibition of Chk2 majorly contributes to the resistance for 5FU-based chemotherapy. The formation of a p21–Chk2 complex disrupts the function of Chk2-mediated apoptosis induction by promoting the nuclear export of both proteins. Screening for the subcellular localization of p21 in patient tumor samples could provide information about possible resistance development under 5FU-based therapy and has to be further evaluated in a clinical setting.

## Supplementary Materials

The following are available online at http://www.mdpi.com/2072-6694/10/10/373/s1, Figure S1: Verification of p-p21T145 antibody for Western Bloting analysis, Figure S2: Localization of p21 in 5FU-resistant HT29 and SW837 cells, Figure S3: Transfection efficiency, Figure S4: Effect of hyperphosphorylated AKT on cellular kinase profiles in HCT116, Figure S5: Interaction between p21 and Chk2 proteins, Figure S6: Interaction between p21 and Chk2 proteins.
