# Supplementary material for: Cytoplasmic p21 Mediates 5-Fluorouracil Resistance by Inhibiting Pro-Apoptotic Chk2
Source: Cancers (Basel). 2018 Oct 9;10(10):373. doi: 10.3390/cancers10100373 (PMC6210175; doi:10.3390/cancers10100373)
Supplement: Supplementary file 1 [file cancers-10-00373-s001.pdf]

Supplementary materials

Cytoplasmic p21 Mediates 5-Fluorouracil Resistance by Inhibiting Pro-Apoptotic Chk2

Arnatchai Maiuthed, Chuanpit Ninsontia, Katharina Erlenbach-Wuensch, Benardina Ndreshkjana, Julienne K. Muenzner, Aylin. Caliskan, Husayn Ahmed P., Chatchai Chaotham, Arndt Hartmann, Adriana Vial Roehe, Vijayalakshmi Mahadevan, Pithi Chanvorachote and Regine Schneider-Stock

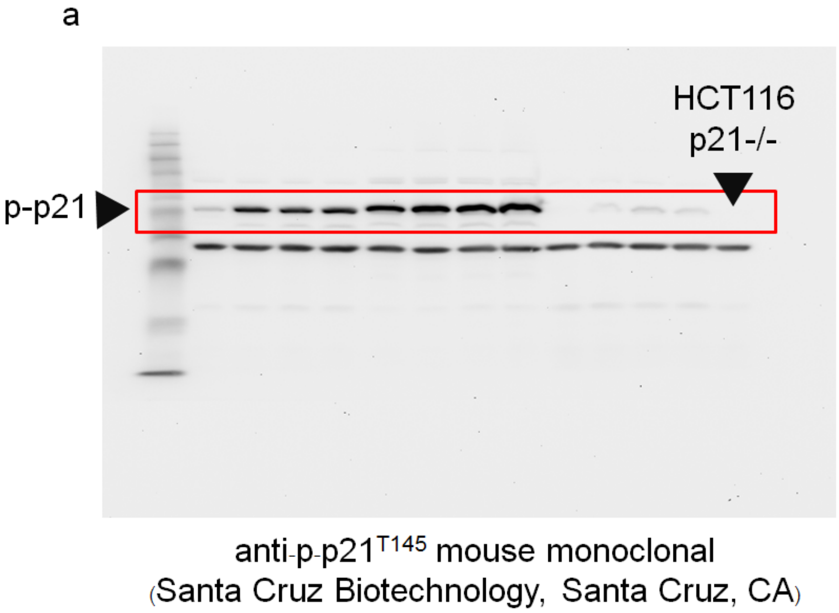

Figure S1. Verification of p-p21^{T145} antibody for Western Bloting analysis. (**a**) p-p21^{T145} showed many unspecific signals in Western Blot analysis, HCT116 p21−/− lysates were used to specify the correct p-p21^{T145} signal.

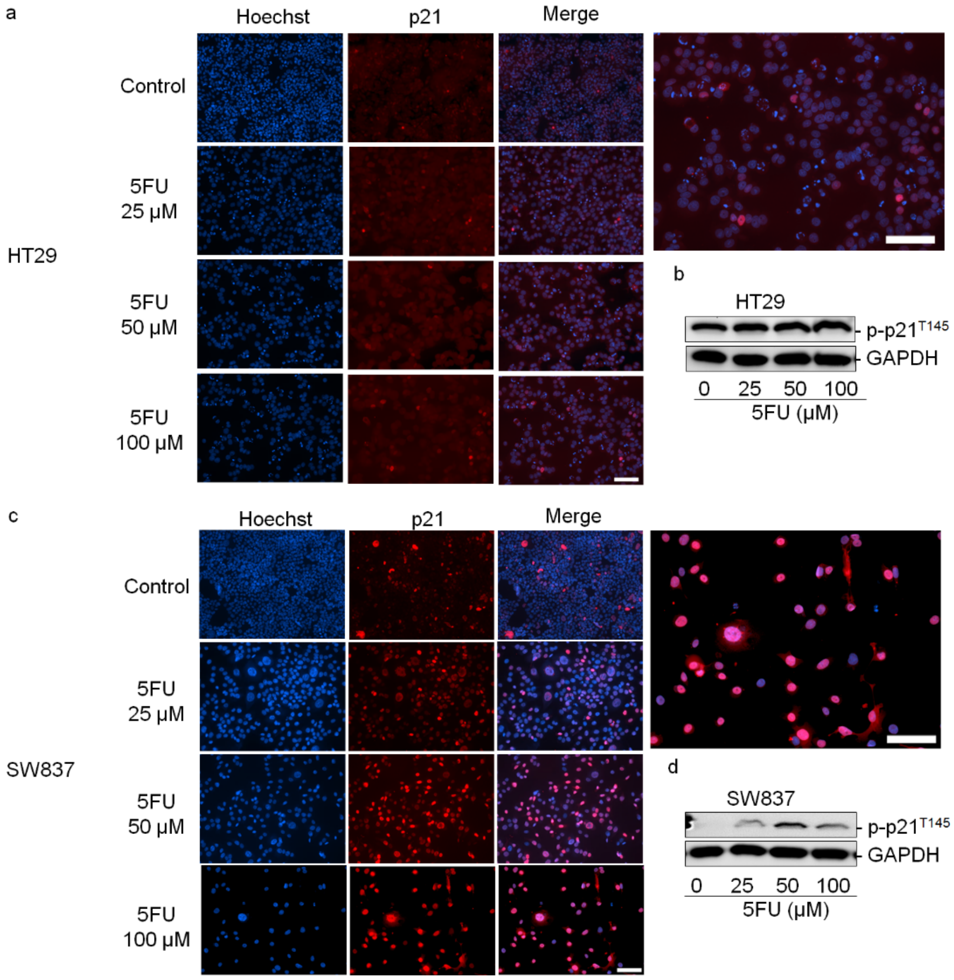

Figure S2. Localization of p21 in 5FU-resistant HT29 and SW837 cells. HT29 cells (**a**,**b**) and SW837 cells (**c**,**d**) were treated with various concentrations of 5FU for 48 h and the expression of p21 was determined by immunofluorescence staining using mouse anti-p21 monoclonal antibodies followed by an Alexa Fluor 555-labeled secondary antibody to visualize p21 expression (red) and cell nuclei (Hoechst 33342, blue) (scale bar: 50 μm). (**a**,**c**) The expression level of phosphorylated-p21 (p-p21^{T145}) in 5FU-resistant HT29 (**b**) and SW837 (**d**) cells was determined by WB analysis. Cells were treated with various concentrations of 5FU for 48 h. After incubation, dead cells were discarded by washing 3 times with PBS and the remaining resistant cells were collected to prepare protein lysates. The expression level of p-p21^{T145} was determined and the blots were re-probed with GAPDH to confirm equal loading of the samples.

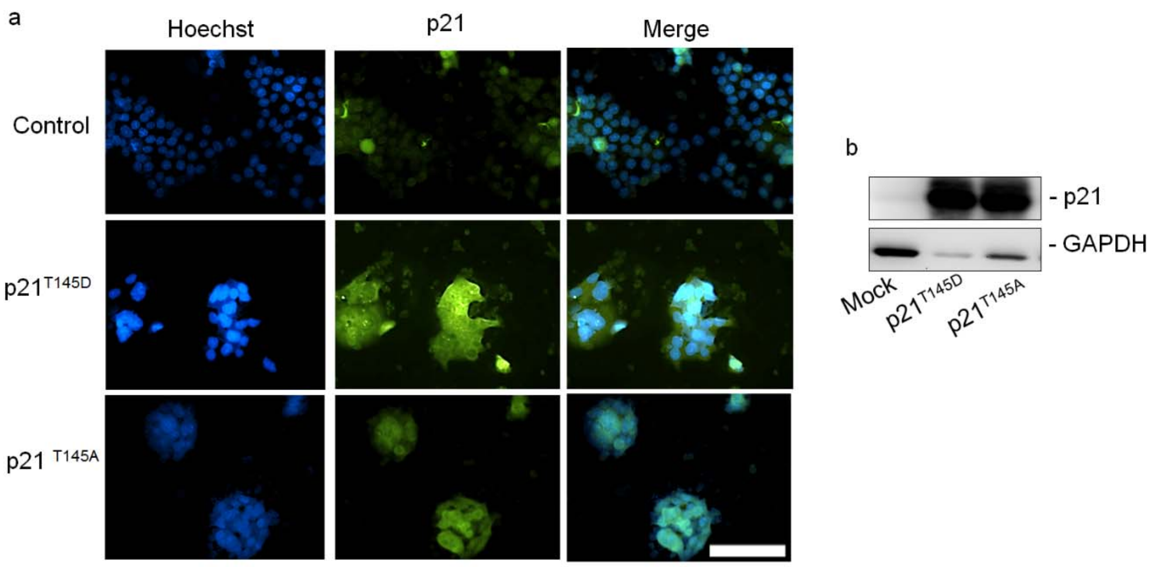

Figure S3. Transfection efficiency. HCT116 cells were transfected with plasmids for the hyperphosphorylated p21^{T145D} and unphosphorylatable p21^{T145A} protein forms. After 24 h of transfection, (**a**) the subcellular localization of p21 was characterized by immunofluorescence staining using mouse anti-p21 monoclonal antibodies followed by Alexa Fluor 488-labeled secondary antibody to visualize p21 expression (green) and cell nuclei (Hoechst 33342, blue), scale bar: 50 μm. (**b**) The expression levels of p21 were quantified by Western Blot analysis after 24 h. Blots were re-probed with GAPDH to confirm equal loading of the samples.

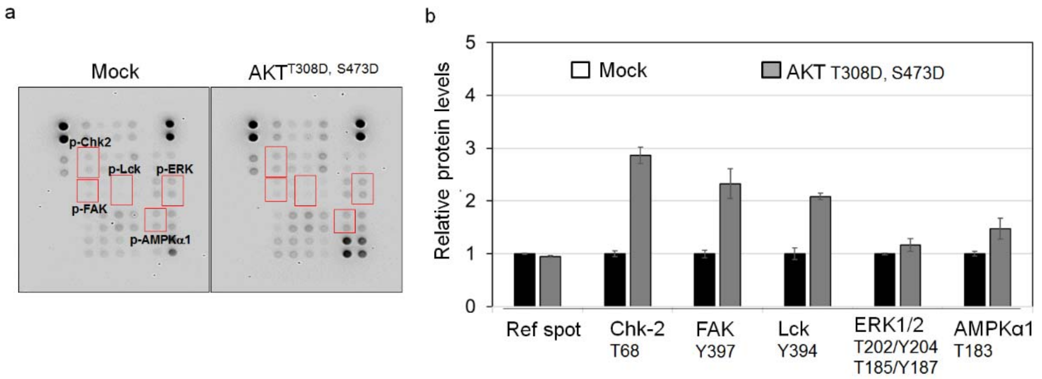

Figure S4. Effect of hyperphosphorylated AKT on cellular kinase profiles in HCT116. (**a**–**b**) Expression levels of phospho-proteins in transfected cells. HCT116 cells were transfected with hyperphosphorylated AKT$^{T308D, S473D}$. After 48 h of transfection, cells lysates were prepared and subjected to the Human Phospho-Kinase Array Kit (R&D systems).

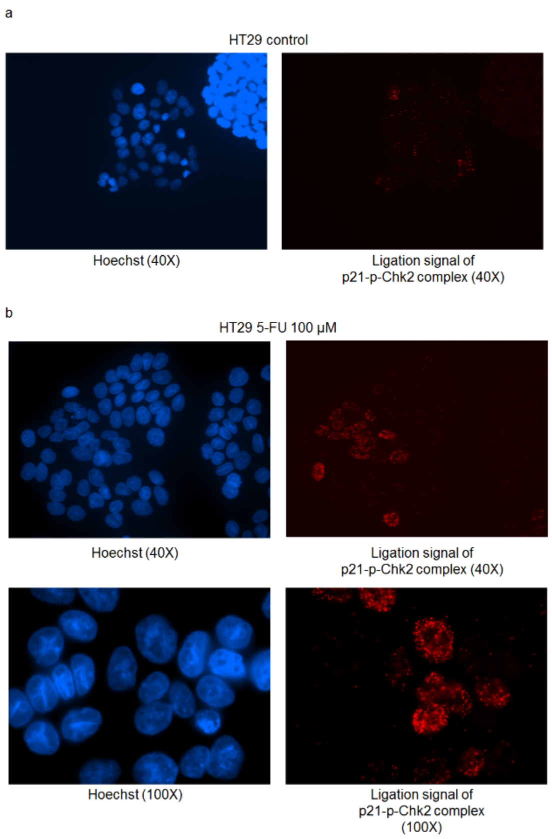

Figure S5. Interaction between p21 and Chk2 proteins. Cells were treated with 100 µM 5FU for 48 h and the protein-protein interaction of p21-p-Chk2^{T68} was analyzed by proximity ligation assay (HT29 cells) (red signals indicate protein-protein interaction between p21 and p-Chk2^{T68}; Fluorescence images of untreated control HT29 cells (**a**, 40× magnification) and 5FU treated HT29 cells (**b**, 40× and 100× magnification).

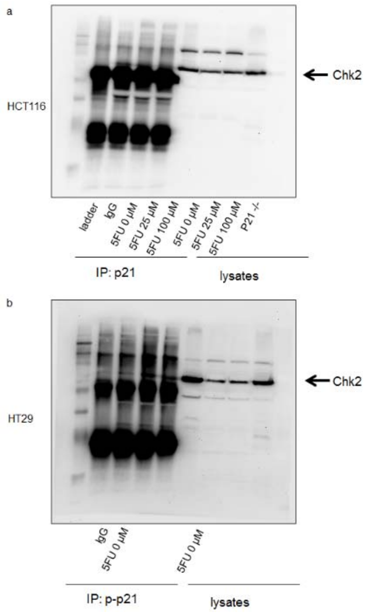

Figure S6. Interaction between p21 and Chk2 proteins. Cells were treated with 25 µM and 100 µM 5FU for 48 h and the protein-protein interaction of p21 and Chk2 (**a**, HCT116 cells) or p-p21^{T145} and Chk2 (**b**, HT29 cells) was analyzed by co-immunoprecipitation assay. Cell lysates were prepared and immunoprecipitated with p21 and p-p21 antibody, respectively. The resulting immunocomplexes were then analyzed for Chk2 by Wetsern Bloting using a Chk2 antibody.
